# An audit: fitness to drive assessment in inpatients of general adult and old age psychiatry

**DOI:** 10.1192/bjo.2021.538

**Published:** 2021-06-18

**Authors:** Ismail Khan, Nneamaka Asiodu, Dr Divyanish, Anum Yaqoob, Hasanain Qureshi

**Affiliations:** 1 Bloxwich Hospital; 2 Bushey Fields Hospital; 3 Heath Lane hospital; 4 Dorothy Pattison Hospital

## Abstract

**Aims:**

To determine if fitness to drive is assessed on admission and discharge, if applicable, and for this to be documented during clerking and on discharge notifications.

To determine if patients are being educated about the impact of their condition on the ability to safely drive.

To ascertain if patients are aware of the duty to inform the DVLA if they for any reason are not fit to drive.

**Background:**

Risk factors include social, behavior and iatrogenic factors such as social withdrawal, increased likelihood of substance abuse and side effects of anti-psychotic medication.

**Method:**

This trust wide audit involved the random sampling of a total of 71 case notes, 4 case notes per Consultant team in general adult psychiatry and old age psychiatry across Dudley and Walsall sites (total of 3 sites). A data collection tool was developed and included relevant questions regarding fitness to drive. Data were collected between October and December 2019.

**Result:**

18/49 patients had physical health screening prior to medication initiation.

**Conclusion:**

An important aspect of good medical practice is to educate patients about their condition, this includes their fitness to drive as this can be affected both by their diagnosis and medication. It is clear that clinicians also need to be educated about this responsibility to ensure assessment is performed especially on inpatient discharge.

